# New Insight Into the Diversity of SemiSWEET Sugar Transporters and the Homologs in Prokaryotes

**DOI:** 10.3389/fgene.2018.00180

**Published:** 2018-05-22

**Authors:** Baolei Jia, Lujiang Hao, Yuan Hu Xuan, Che Ok Jeon

**Affiliations:** ^1^School of Bioengineering, Qilu University of Technology (Shandong Academy of Sciences), Jinan, China; ^2^Department of Life Science, Chung-Ang University, Seoul, South Korea; ^3^College of Plant Protection, Shenyang Agricultural University, Shenyang, China

**Keywords:** SemiSWEET, sugar transporter, diversity, evolution, prokaryote

## Abstract

Sugars will eventually be exported transporters (SWEETs) and SemiSWEETs represent a family of sugar transporters in eukaryotes and prokaryotes, respectively. SWEETs contain seven transmembrane helices (TMHs), while SemiSWEETs contain three. The functions of SemiSWEETs are less studied. In this perspective article, we analyzed the diversity and conservation of SemiSWEETs and further proposed the possible functions. 1,922 SemiSWEET homologs were retrieved from the UniProt database, which is not proportional to the sequenced prokaryotic genomes. However, these proteins are very diverse in sequences and can be classified into 19 clusters when >50% sequence identity is required. Moreover, a gene context analysis indicated that several SemiSWEETs are located in the operons that are related to diverse carbohydrate metabolism. Several proteins with seven TMHs can be found in bacteria, and sequence alignment suggested that these proteins in bacteria may be formed by the duplication and fusion. Multiple sequence alignments showed that the amino acids for sugar translocation are still conserved and coevolved, although the sequences show diversity. Among them, the functions of a few amino acids are still not clear. These findings highlight the challenges that exist in SemiSWEETs and provide future researchers the foundation to explore these uncharted areas.

## Introduction

Membrane transporters control the influx and efflux of a wide variety of molecules, such as sugars, amino acids, organic acids, nitrate, and ammonium. Sugars will eventually be exported transporters (SWEETs) mediate the uptake or efflux of various mono and disaccharides across the plasma membrane by allowing solutes to permeate across biological membranes down a concentration gradient (Chen, 2014). Eukaryotic SWEETs are generally composed of seven transmembrane helices (TMHs) that contain a pair of three TM repeats, which are connected by an additional helix, while SemiSWEETs, the homologs of SWEETs in prokaryotes, contain three TMHs (Xuan et al., 2013). The structure of the OsSWEET2b from rice (*Oryza sativa*) has been determined, and it showed that the proteins form a homotrimeric complex (Tao et al., 2015). Meanwhile, the structures of the prokaryotic SemiSWEETs from *Leptospira biflexa* (LbSemiSWEET), *Escherichia coli* (EcSemiSWEET), *Thermodesulfovibrio yellowstonii* (TySemiSWEET), and *Vibrio* sp. (VsSemiSWEET) form symmetrical, parallel dimers, thereby generating the translocation pathway (Wang et al., 2014; Xu et al., 2014; Lee et al., 2015; Latorraca et al., 2017). The function of SWEETs in plants has been well-studied, and they play key roles in sugar translocation, nectar secretion, pollen nutrition, seed filling, and pathogen nutrition (Feng and Frommer, 2015). In *Arabidopsis thaliana* containing 17 SWEETs, AtSWEET1/4/5/7/8/13 mediate the efflux of glucose, AtSWEET11/12 function as fructose transporters, and AtSWEET17 permeates sucrose (Feng and Frommer, 2015). However, the function of SemiSWEETs are seldom studied. BjSemiSWEET (from *Bradyrhizobium japonicum*) can mediate cellular sucrose uptake and efflux (Xuan et al., 2013). EcSemiSWEET showed the sucrose transport activity, but sucrose might not be the physiological substrate because the uptake is unusually slow (Lee et al., 2015). A glucose uptake-deficient *E. coli* strain expressing wild-type LbSemiSWEET showed a significantly higher glucose uptake than the controls, indicating LbSemiSWEET can function as a glucose transporter (Xu et al., 2014). Except for the three SemiSWEETs, little is known about the functions of other homologs.

Our recent study indicated that gene duplication and gene fusion are important factors driving the evolution of SWEETs, while the sequences of SemiSWEETs showed more diversity than SWEETs in plants (Jia et al., 2017b). In this perspective paper, we retrieved the sequences of SemiSWEETs from the UniProt database and analyzed them using several bioinformatics methods (the methods used in this study are listed in the Supplementary Materials). Based on the global sequence analysis, we give clues to the diversity, function, and conservation of SemiSWEETs in prokaryotes.

## Distribution and Diversity of SemiSweets in Prokaryotes

**FIGURE 1:**
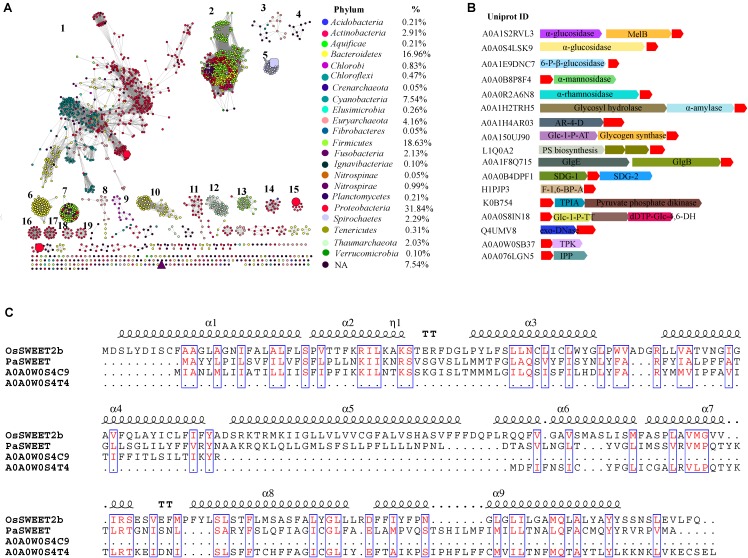
Global view of sequence diversity of SemiSWEETs. **(A)** The protein sequence similarity network (SSN) of SemiSWEETs from prokaryotes. 1,922 protein sequences were analyzed by means of an SSN with an e-value threshold of 10^-25^ (>50% sequence identity). Each node represents one protein. Edges are shown with BLASTP e-values below the indicated cut-off. A cluster was sequentially labeled if there were more than 10 nodes in it. Nodes from the same phyla in the global network have the same color. The colors corresponding to each class and protein percentage in each class are listed at the bottom. BjSemiSWEET (hexagon), LbSemiSWEET (rectangle), EcSemiSWEET (octagon), TySemiSWEET (triangle), and VsSemiSWEET (cycle) are enlarged. **(B)** Schematic view of the genomic context of SemiSWEETs. The UniProt IDs of the SemiSWEETs are listed on the left. The SemiSWEETs or SWEETs are shown in red. The annotated names of other genes are shown on the genes. The abbreviations are AR-4-D, D-arabinitol 4-dehydrogenase; Glc-1-P-AT, glucose-1-phosphate adenylyltransferase; PS biosynthesis, polysaccharide synthesis; GlgB, 1,4-α-glucan branching enzyme; GlgE, α-1,4-glucan:maltose-1-phosphate maltosyltransferase; SDG, saccharopine dehydrogenase; F-1,6-BP-A, fructose-1,6-bisphosphate aldolase; TPIA, triosephosphate isomerase; Glc-1-P-TT, glucose-1-phosphate thymidylyltransferase; dTDP-Glc-4,6-DH, dTDP-glucose 4,6-dehydratase; TPK, thiamin pyrophosphokinase; IPP, inositol-phosphate phosphatase. **(C)** Sequence alignment of OsSWEET2b, PaSWEET, A0A0W0S4C9, and A0A0W0S4T4. Identical and similar amino acids are shown in red and boxed.

Sugars will eventually be exported transporters and SemiSWEETs contain the domain harboring the PQ-loop repeat. This domain is also named MtN3_slv, because the first gene in the family, *MtN3*, was identified in the legume *Medicago truncatula* (Gamas et al., 1996). Later, another gene, *saliva*, was identified in *Drosophila*, which had a high transcript level in the salivary gland (Artero et al., 1998; Yuan et al., 2014). We collected 1,995 homologs of SemiSWEETs and/or SWEETs from the UniProt protein database in prokaryotes. These proteins were annotated by Pfam protein database^1^ and 1, 922 were screened to contain at least one MtN3_slv motif (PF03083) (Supplementary Data Sheet 1). The sequence similarity networks (SSNs) for the 1,922 sequences were constructed using the e-value cut-offs of 10^-10^, at which >30% sequence identity was required to draw an edge between nodes (Jia et al., 2017a). At this e-value threshold, almost all of the nodes are located in one cluster, and the five studied SemiSWEETs (BjSemiSWEET, LbSemiSWEET, EcSemiSWEET, TySemiSWEET, and VsSemiSWEET) are included in the cluster (Supplementary Figure 1). As the e-value threshold stringency is decreased to 10^-25^ (sequence identity required to draw an edge is decreased to > ∼50%), BjSemiSWEET transporting sucrose and LbSemiSWEET transporting glucose can be separated into different clusters, and the 1,922 proteins can be segregated into 19 clusters containing 10 or more members (**Figure 1A**). Meanwhile, another three studies of SemiSWEETs were also separated. Among them, BjSemiSWEET has the largest number of homologs, followed by LbSemiSWEET and VsSemiSWEET. EcSemiSWEET and TySemiSWEET seldom have homologs sharing >50% identity. Sequence alignments of the five proteins indicated that BjSemiSWEET shared 44% identity to TySemiSWEET (the highest value among them), while VsSemiSWEET only have 15% identity to LbSemiSWEET (the lowest one) (Supplementary Figure 2). This analysis suggested that SemiSWEETs are very heterogeneous in sequences. Furthermore, only a minority of clusters (3/19) have been annotated in Swiss-Prot or in the literature, suggesting that the proteins in another 16 clusters may transport different substrates.

Considering the explosive growth of bacterial genome sequences and the fact that only few SemiSWEETs are found in the databases, the nodes were painted by taxonomic classification to explore the occurrence of the transporters. SemiSWEETs can be found in both archaea and bacteria. In archaea, members of SemiSWEETs (clusters 3 and 12) were found only in two of five phyla in the domain (*Euryarchaeota* and *Thaumarchaeota*). In bacteria, the proteins were distributed in 20 phyla. These proteins are mainly found in *Proteobacteria* (33.00%), *Firmicutes* (18.10%), *Bacteroidetes* (16.35%), and *Cyanobacteria* (7.47%). The presence of SemiSWEETs in other classes is limited. Notably, almost all the individual clusters contain proteins belonging to the same phylum, or the proteins in the same phylum are close to each other within one cluster. The same phenomena suggested that the horizontal gene transfer among different phyla seldom occurred during the evolution and distribution of SemiSWEETs.

## Gene Context Analysis

Gene context assays can detect the functional association of proteins, such as interaction subunits, members of the same pathway and an enzyme and its regulator (Kanehisa and Bork, 2003). To better understand the role of SemiSWEETs, a gene context analysis was performed to determine their potential operonic associations, and the gene clusters related to sugar metabolisms were listed (**Figure 1B**). α-glucosidases can breakdown starch and/or disaccharides to glucose. In *Citrobacter freundii* and *Nitrospira nitrificans*, the SemiSWEET genes (UniProt IDs: A0A0S4LSK9 and A0A1S2RVL3) are clustered with the α-glucosidases. In *C. freundii*, a melibiose:sodium transporter (MelB) is further associated with the SemiSWEET and α-glucosidase gene. Similarly, the SemiSWEETs from *Streptococcus* sp. (UniProt ID: A0A1E9DNC7), *Vibrio ishigakensis* (UniProt ID: A0A0B8P8F4), *Lactobacillus vaccinostercus* (UniProt ID: A0A0R2A6N8) and *Nitrosomonas communis* (UniProt ID: A0A1H2TRH5) are associated with 6-phospho-β-glucosidase, α-mannosidase, α-rhamnosidase, and α-amylase, respectively, indicating these SemiSWEETs may be involved in the transport of the corresponding sugars. In *Lonsdalea quercina*, the SemiSWEET gene (UniProt ID: A0A1H4AR03) is tightly associated with the gene encoding D-arabinitol 4-dehydrogenase, which catalyses the production of D-xylulose. The association between the two genes implies that the SemiSWEET can transport D-xylulose. Curiously, SemiSWEETs are also associated with the genes related to sugar synthesis. In *Bradyrhizobium* sp. AT1, *Capnocytophaga* sp., and *Chloroflexi bacterium*, three SemiSWEET genes (UniProt IDs: A0A150UJ90, L1Q0A2, and A0A1F8Q715) are located downstream of the genes involved in the synthesis of sugar, such as glycogen synthase and other polysaccharide biosynthesis protein, implying that the three SemiSWEETs may be involved in the metabolism of sugar synthesis. Furthermore, the SemiSWEETs are also in the same cluster with the genes involved in other small molecules metabolism. For example, a SemiSWEET (UniProt ID: A0A0B4DPF1) in *Leisingera* sp. ANG1 is located between the genes encoding the two subunits of saccharopine dehydrogenase, an enzyme involved in the metabolism of the lysine and pyridine nucleotide, suggesting the SemiSWEET may also be involved in the process (Andi et al., 2007). Fructose-1,6-bisphosphate (FBP) aldolases reversibly cleave FBP into two triose phosphates, glycerone phosphate and D-glyceraldehyde 3-phosphate. In *Eubacterium infirmum*, a SemiSWEET (UniProt ID: H1PJP3) is clustered with the gene. In *Nitrosopumilus koreensis*, the SemiSWEET (UniProt ID: K0B754) is clustered with triosephosphate isomerase and pyruvate phosphate dikinase, both of which are involved in the metabolism of C3 molecules. These analyses suggest that the SemiSWEETs may participate in the transport of related molecules. SemiSWEETs may also be involved in the metabolism of nucleotide sugars. In *Omnitrophica* WOR_2, the SemiSWEET (UniProt ID: A0A0S8IN18) is found in gene clusters together with genes encoding glucose-1-phosphate thymidylyltransferase and dTDP-glucose 4,6-dehydratase. In *Rickettsia felis*, the SemiSWEET (UniProt ID: Q4UMV8) is clustered with a gene encoding exodeoxyribonuclease III. Finally, the SemiSWEETs may also transport secondary metabolites. A thiamin pyrophosphokinase participating in thiamine metabolism is associated with the SemiSWEET (UniProt ID: Q4UMV8) in *Legionella cherrii*, and a gene encoding an inositol-phosphate phosphatase involved in inositol metabolism is also found with the SemiSWEET (UniProt ID: A0A076LGN5) in *Methanocaldococcus bathoardescens*. Based on the gene context assay, we propose that SemiSWEETs may participate in several metabolic pathways and transport many molecules.

## Gene Fusion of MtN3-Like Proteins

Pfam database groups PQ-loop repeat proteins and SWEETs (MtN3_slv) into the same MtN3-like clan (CL0141) (Feng and Frommer, 2016). PQ-loop-containing proteins transport cystine, lysine, or arginine across the membrane (Liu et al., 2012). In this study, we identified 20 proteins with seven TMHs that harbor at least one MtN3_slv motif in bacteria. These proteins can be classified into three clusters based on a phylogenetic analysis (Supplementary Figure 3). The proteins in cluster 2 and cluster 3 only contain one MtN3_slv motif even they have seven TMHs (Supplementary Data Sheet 1). Most members in cluster 1 contain one MtN3_slv motif and another PQ-loop repeat. We chose one protein from *Pseudoalteromonas* sp. SCSIO_11900 (PaSWEET, UniProt ID: Z9K5C6) in cluster 1 to align with the OsSWEET2b. The PaSWEET showed low identity to OsSWEET2b (25%), but the amino acids in the TMHs were conserved (**Figure 1C**). Further alignment of PaSWEET with the homologs in UniProt database showed that the N-terminal 3-TMHs segments of PaSWEET had 33% identity to a protein with PQ-loop repeat from *Legionella brunensis* (UniProt ID: A0A0W0S4C9). In the genome of *L. brunensis*, another PQ-loop repeat protein (UniProt ID: A0A0W0S4T4) showed 42% identity to the C-terminal three-TMHs segments of PaSWEET. Furthermore, the genes encoding the two proteins were located close to each other in the genome the *L. brunensis*, which is similar to OsSWEET6a and OsSWEET6b in the genome of rice (Jia et al., 2017b). Based on the sequence identity and the increased number of TMH units, we proposed that PQ-loop proteins and SWEETs may derive from a potential common ancestor, and gene duplication/fusion may occur during the evolution of these proteins.

## Consensus and Coevolution of Amino Acids in SemiSweets

**FIGURE 2:**
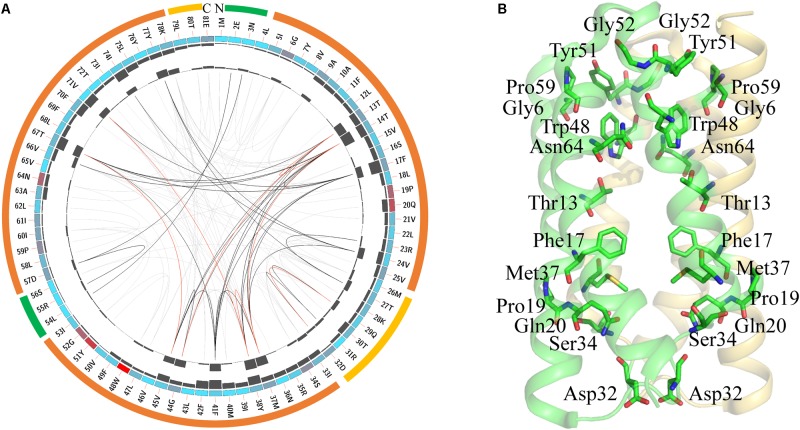
Conserved amino acid residues in SemiSWEETs represented using LbSemiSWEET as a reference sequence. **(A)** Network analysis of conserved and coevolving residues. The circular network shows the connectivity of coevolving residues. The first circle represents the TM helices (orange), the intrafacial region (yellow), and the extrafacial region (green). The labels in the second circle indicate the alignment position and the amino acid code of EcSWEET. The colored square boxes of the second circle indicate MSA position conservation (highly conserved positions are in red, whereas less conserved positions are in blue). The third and fourth circles show the proximity mutual information (MI) and cumulative MI (cMI) values as histograms, facing inward and outward, respectively. In the center of the circle, the edges that connect pairs of positions represent a significant MI value (>6.5), highlighted in red lines with higher MI scores (top 5%), black lines (between 70 and 95%), and gray lines (the remaining 70%), as defined by MISTIC. **(B)** Cartoon diagram of EcSWEET (PDB ID: 4qnc) with the conserved residues.

SemiSWEETs may be diverse in transporting different molecules. To examine if the protein sequences are conserved during evolution, the consensus sites of the SemiSWEETs were analyzed by multiple sequence alignments (MSAs). The protein sequence of LbSemiSWEET from with UniProt ID B0SR19 was used as the reference sequence for MSAs, and the conservation of the residues are shown in **Figure 2A**. The highly conserved residues were mapped on the structure of LbSemiSWEET (**Figure 2B**) and further analyzed by amino acids coevolution (Supplementary Figure 4).

LbSemiSWEET forms a homo-dimer structure, and each protomer is composed of three TMHs (Xu et al., 2014). In the TMH1, the signature PQ motif consisting of both Pro and Gln residues is highly conserved (**Figure 2A**). The conserved PQ motif is embedded in the membrane, which is in contrast to the assumption that the motif is positioned in a loop region. The Gln can stabilize the dimer structure, while the Pro induces a kink in the helix, which probably enables the ‘binder clip-like’ motion of the protein (Xu et al., 2014). In EcSemiWEET, the mutation of Pro to Ala dramatically decreases the activity, while Gln replaced by Ala slightly affects the activity (Lee et al., 2015). The PQ motif together with the highly conserved Phe17, Asp32, Ser34, and Met37 form a key cluster for the intracellular gate (**Figure 2B**). However, M39A in LbSemiSWEET had no significant effect on transporter activity, as similar as it is to the M39A mutation in EcSemiSWEET (Xu et al., 2014; Lee et al., 2015). In TMH2, a Trp is the most highly conserved residue in the SemiSWEETs (**Figure 2A**). Trp48, Asn64, and Thr13 are the major constituents of the putative substrate binding pocket of the protein in EsSWEET (**Figure 2B**). In LbSemiSWEET, Trp48, and Asn64 play a particularly critical role in coordinating the substrate (Latorraca et al., 2017). During substrate translocation, the glucose ring was partially sandwiched between the pyrrole rings of Trp48 on each protomer. Asn64 and Thr13 on only one protomer directly coordinate substrate hydroxyl groups (Latorraca et al., 2017). When Trp or Asn was mutated to Ala, the uptake activity of SemiSWEETs from *E. coli* and *L. biflexa* was markedly reduced (Xu et al., 2014; Lee et al., 2015). The other two conserved residues in TMH2 are Tyr51 and Gly52 (**Figure 2A**). In EcSemiSWEET, Tyr53, Arg57, and Asp59 interact with the equivalent residues of the adjacent protomer, and these interactions completely seal off the substrate-binding pocket from the extracellular environment. In LbSemiSWEET, the motion of Tyr51, Ile60, and Phe17 is responsible for opening a pathway to the substrate-binding pocket on the intracellular side, and alanine substitution of each of these three residues decreased the transporting activity (Latorraca et al., 2017). Our results indicated that Tyr51 and Phe17 but not Ile60 are conserved. Meanwhile, Gly6, Gly52, and Pro59 located near the extrafacial gate are also conserved, but their functions have not been examined. Further mapping of the top conserved amino acids in the structure of LbSemiWEET revealed that these amino acids form three conserved clusters, which may have the putative function for the extrafacial gate, substrate binding, and intrafacial gate (**Figure 2B**). Each conserved residue belongs to a cluster: four to the extrafacial gate, three to the substrate binding pocket, and six to the intrafacial gate.

The coevolution of SemiSWEET amino acids was also investigated using MI (**Figure 2A**). Compared with the amino acids of SWEETs (Jia et al., 2017b), only a few pairs of amino acids in SemiSWEET were highly coevolved. The top conserved residues in SWEETs were further considered for a coevolution analysis in order to be illustrated clearly, as shown in Supplementary Figure 4. The top 15 scoring residues formed a connected distance network, indicating these residues also share a significant MI value. Similar to the residues in SWEETs, we propose here that the conserved and coevolving amino acids in SemiSWEET also play important roles in both binding and transporting sugars.

## Discussion and Future Outlook

The large reduction in the price of sequencing as a result of technical developments subsequently increases microbiological knowledge. The number of sequenced prokaryotic genomes in the NCBI database exceeds 120,000 and grows daily (Land et al., 2015; Zuo and Hao, 2017). The total number of semiSWEET genes in prokaryotes is not proportional to the sequenced genomes, as we only retrieved <2,000 semiSWEET homologs in the database. The analysis indicated that SemiSWEETs are distributed among archaea and bacteria but are sparse; that is, only few prokaryotes have a SemiSWEET. The limited number of SemiSWEET homologs suggested that the functions of SemiSWEETs in prokaryotes are not as important as the SWEETs in eukaryotes. In plants, SWEETs are involved in sugar efflux from phloem parenchyma cells, loading in nectar secretion, seed filling, pollen nutrition, and pathogen susceptibility (Feng and Frommer, 2015). Little is known about the physiological function of SWEETs in Metazoa. However, mutations of the SWEET gene in the sea squirt caused defects early in development (Hamada et al., 2005). Compared with the eukaryotic SWEETs, nothing is known about the physiological function of prokaryotic SemiSWEETs until now. Xuan et al. (2013) tested eight SemiSWEETs from bacteria, but only BjSemiSWEET mediates sucrose uptake in human HEK293T cells and efflux in *Synechococcus elongatus*. However, the physiological role of BjSemiSWEET is still not proven. Our gene neighbor analysis indicated that several SemiSWEETs are located in the operons related to sugar or other carbohydrate metabolism, such as starch, mannose, rhamnose, xylulose, nucleotide, and inositol. Generally, the codirectional gene neighbor is a good indicator of functional relevance in prokaryotes, and it allows biologically relevant signals to be discerned from background noise (Korbel et al., 2004). The diverse gene neighbors of SemiSWEETs suggested that SemiSWEETs may transport diverse substrates and play diverse physiological roles in different organisms. A mutation analysis of these SemiSWEETs can help determine their native substrates and elucidate their physiological roles in organisms.

Gene duplication and gene fusion are important factors driving the evolution of SWEETs (Jia et al., 2017b). In plants, an ExtraSWEET protein from *Vitis vinifera*, consisting of 14 TMHs, may be fused by two SWEETs (Patil et al., 2015). The SuperSWEETs from oomycetes contain 25 TMHs, which may represent four duplications of SWEET (Jia et al., 2017b). In this study, we showed that two PQ-loop proteins may also be duplicated and further fused the genome of bacteria. The duplication and fusion of the proteins in MtN3-like clan could be an efficient way to create a functional translocation pore evolutionally, but whether and how they are formed from the fusion remain to be further explored.

Even the SemiSWEETs are diverse in sequences and potentially in function, and the structure study in all four SemiSWEETs showed that the dimer is the fundamental structure of SemiSWEETs. The crystals structures together with molecular dynamics simulations revealed the mechanism for glucose translocation and the function of the amino acids during translocation. These amino acids are generally highly conserved and coevolved based on the MSA and MI from 1,922 sequences, such as the Pro and Gln in the PQ motif and Trp and Asn in the binding pocket. However, several amino acids playing important roles in translocation are not conserved, such as Arg55 and Asp57 near the extracellular surface, Ile60 above the glucose binding pocket, and Tyr38 and Phe41 near the intracellular surface. The mutation of Asp57 to Ala increased the affinity to glucose (Latorraca et al., 2017). We proposed that these amino acids are responsible for specific recognition and translocation of glucose. The mutation of amino acids in the positions may lead to neo-functionalization (i.e., new function) (Jia et al., 2017b). On the other hand, the function of several highly conserved amino acids is less studied, including two Gly in the extracellular surface and the Asp in the intracellular surface. Further mutagenesis or other studies such as nuclear magnetic resonance may elucidate the roles of these conserved amino acids.

## Author Contributions

BJ and CJ conceived, designed the data analysis, and wrote the manuscript. LH and YX helped to analyze the data.

## Conflict of Interest Statement

The authors declare that the research was conducted in the absence of any commercial or financial relationships that could be construed as a potential conflict of interest.

## Footnotes

1 http://pfam.xfam.org/
