# Supplementary material for: New Insight Into the Diversity of SemiSWEET Sugar Transporters and the Homologs in Prokaryotes
Source: Front Genet. 2018 May 22;9:180. doi: 10.3389/fgene.2018.00180 (PMC5972207; doi:10.3389/fgene.2018.00180)
Supplement: Supplementary file 1 [file Presentation_1.pdf]

New insight into the diversity of SemiSWEET sugar transporters and the homologs in prokaryotes

Baolei Jia[1,2,*], Lujiang Hao[1], Yuan Hu Xuan[3], and Che Ok Jeon[2,*]

[1]School of Bioengineering, Qilu University of Technology (Shandong Academy of Sciences), Jinan, China

[2]Department of Life Science, Chung-Ang University, Seoul, Republic of Korea

[3]College of Plant Protection, Shenyang Agricultural University, Shenyang, China

Corresponding authors

baoleijia@cau.ac.kr (BJ)

cojeon@cau.ac.kr(COJ)

Running title: A comprehensive analysis of SemiSWEETs

Methods

Collection of SemiSWEETs from prokaryotes

The complete amino acid sequences of experimentally characterized SemiSWEETs were collected from the literature, with a publication cutoff of 2017. These sequences were used as a bait and searched in the Uniprot database using BLAST with e-value cutoff of e-^{10}. The sequences were also retrieved from the InterPro webpage (http://www.ebi. ac.uk/interpro/; release 66.0) (Mitchell et al., 2015). The proteins identified in the database are listed in Supplementary Dataset.

Construction of SSNs and gene context assay

SSNs were constructed using the Enzyme Function Initiative-Enzyme Similarity Tool (EFI-EST) (Gerlt et al., 2015) and were visualized by Cytoscape 3.3 (Shannon et al., 2003). The input sequences were retrieved from the BLAST results from the UniProt database and from the Interpro database. Each node in the network indicates a protein, and the edge indicates that the two nodes share significant similarity with an e-value less than the selected cutoff. The gene context of SemiSWEETs were analyzed by EFI--genome neighborhood networks tool (Gerlt, 2017).

Multiple sequence alignments (MSAs) and coevolving protein residues

MSAs of protein sequences were performed in the Clustal Omega (version 2) software (Sievers et al., 2011). Analysis of coevolving residues was conducted using mutual information (MI) between two positions in an MSA. Phylogenetic trees were constructed in MEGA7 using the maximum likelihood (ML) and the bootstrap test carried out with 1000 iterations (Baolei Jia, 2015;Kumar et al., 2016). MI reflects the extent to which knowing an amino acid at one position can predict the amino acid identity at the other position. MI was calculated between pairs of columns in the MSA, using the MISTIC approach and web server (Simonetti et al., 2013).

References:

Baolei Jia, Z.L., Jinliang Liu, Ying Sun, Xiaomeng Jia, Yuan Hu Xuan, Jiayan Zhang, Che Ok Jeon (2015). A Zinc-Dependent Protease AMZ-tk from a Thermophilic Archaeon is a New Member

of the Archaemetzincin Protein Family. *Front Microbiol* 6, 1380,

Gerlt, J.A. (2017). Genomic Enzymology: Web Tools for Leveraging Protein Family Sequence–Function Space and Genome Context to Discover Novel Functions. *Biochemistry* 56, 4293-4308.10.1021/acs.biochem.7b00614

Gerlt, J.A., Bouvier, J.T., Davidson, D.B., Imker, H.J., Sadkhin, B., Slater, D.R., and Whalen, K.L. (2015). Enzyme Function Initiative-Enzyme Similarity Tool (EFI-EST): A web tool for generating protein sequence similarity networks. *Biochim Biophys Acta* 1854, 1019-1037.10.1016/j.bbapap.2015.04.015

Kumar, S., Stecher, G., and Tamura, K. (2016). MEGA7: Molecular Evolutionary Genetics Analysis version 7.0 for bigger datasets. *Molecular Biology and Evolution*.10.1093/molbev/msw054

Mitchell, A., Chang, H.Y., Daugherty, L., Fraser, M., Hunter, S., Lopez, R., Mcanulla, C., Mcmenamin, C., Nuka, G., Pesseat, S., Sangrador-Vegas, A., Scheremetjew, M., Rato, C., Yong, S.Y., Bateman, A., Punta, M., Attwood, T.K., Sigrist, C.J., Redaschi, N., Rivoire, C., Xenarios, I., Kahn, D., Guyot, D., Bork, P., Letunic, I., Gough, J., Oates, M., Haft, D., Huang, H., Natale, D.A., Wu, C.H., Orengo, C., Sillitoe, I., Mi, H., Thomas, P.D., and Finn, R.D. (2015). The InterPro protein families database: the classification resource after 15 years. *Nucleic Acids Res* 43, D213-221.10.1093/nar/gku1243

Shannon, P., Markiel, A., Ozier, O., Baliga, N.S., Wang, J.T., Ramage, D., Amin, N., Schwikowski, B., and Ideker, T. (2003). Cytoscape: a software environment for integrated models of biomolecular interaction networks. *Genome Res* 13, 2498-2504.10.1101/gr.1239303

Sievers, F., Wilm, A., Dineen, D., Gibson, T.J., Karplus, K., Li, W., Lopez, R., Mcwilliam, H., Remmert, M., Soding, J., Thompson, J.D., and Higgins, D.G. (2011). Fast, scalable generation of high-quality protein multiple sequence alignments using Clustal Omega. *Mol Syst Biol* 7, 539.10.1038/msb.2011.75

Simonetti, F.L., Teppa, E., Chernomoretz, A., Nielsen, M., and Marino Buslje, C. (2013). MISTIC: Mutual information server to infer coevolution. *Nucleic Acids Res* 41, W8-14.10.1093/nar/gkt427

Supplementary Fig. 1

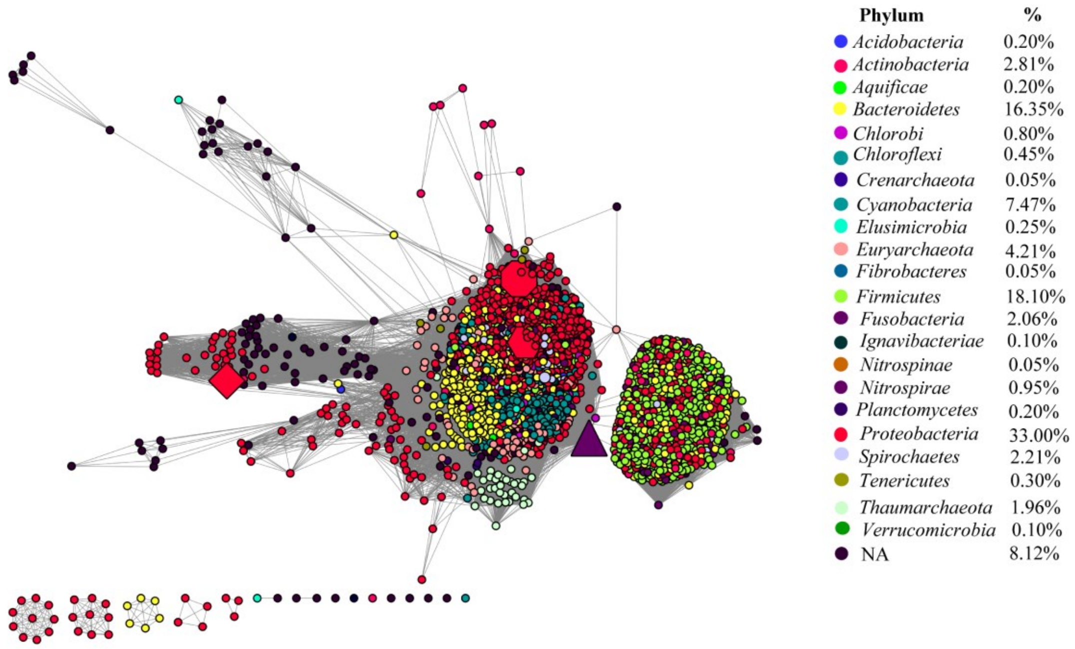

Supplementary Fig. 1. The protein sequence similarity network (SSN) of SemiSWEETs from prokaryotes. 1,995 protein sequences were analyzed by means of an SSN with an e-value threshold of 10^{-10} (>30% sequence identity). Each node represents one protein. Edges are shown with BLASTP e-values below the indicated cutoff. A cluster was sequentially labeled if there were more than 10 nodes in it, and each cluster was assigned a different color. Nodes from the same phyla in the global network have the same color. The colors corresponding to each class and protein percentage in each class are listed at the bottom. BjSemiSWEET, LbSemiSWEET, EcSemiSWEET, TySemiSWEET, and VsSemiSWEET are enlarged.

Supplementary Fig. 2.

A

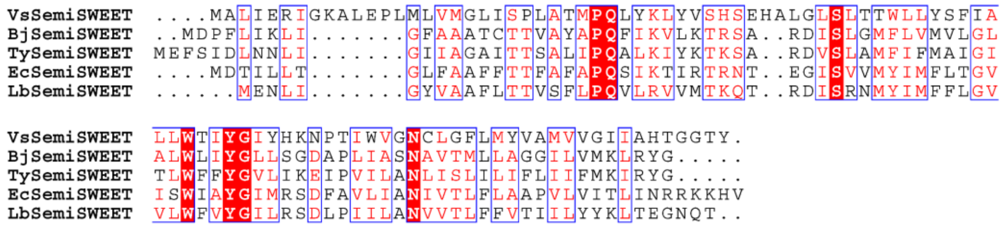

B

Protein name	VsSemiSWEET	BjSemiSWEET	TySemiSWEET	EcSemiSWEET	LbSemiSWEET
VsSemiSWEET	100	22.62	22.62	17.05	15.29
BjSemiSWEET		100	44.19	36.9	42.68
TySemiSWEET			100	27.38	40.24
EcSemiSWEET				100	42.35
LbSemiSWEET					100

Supplementary Fig. 2. Sequence alignments (A) and percent identity (B) of five SemiSWEETs.

Supplementary Fig. 3.

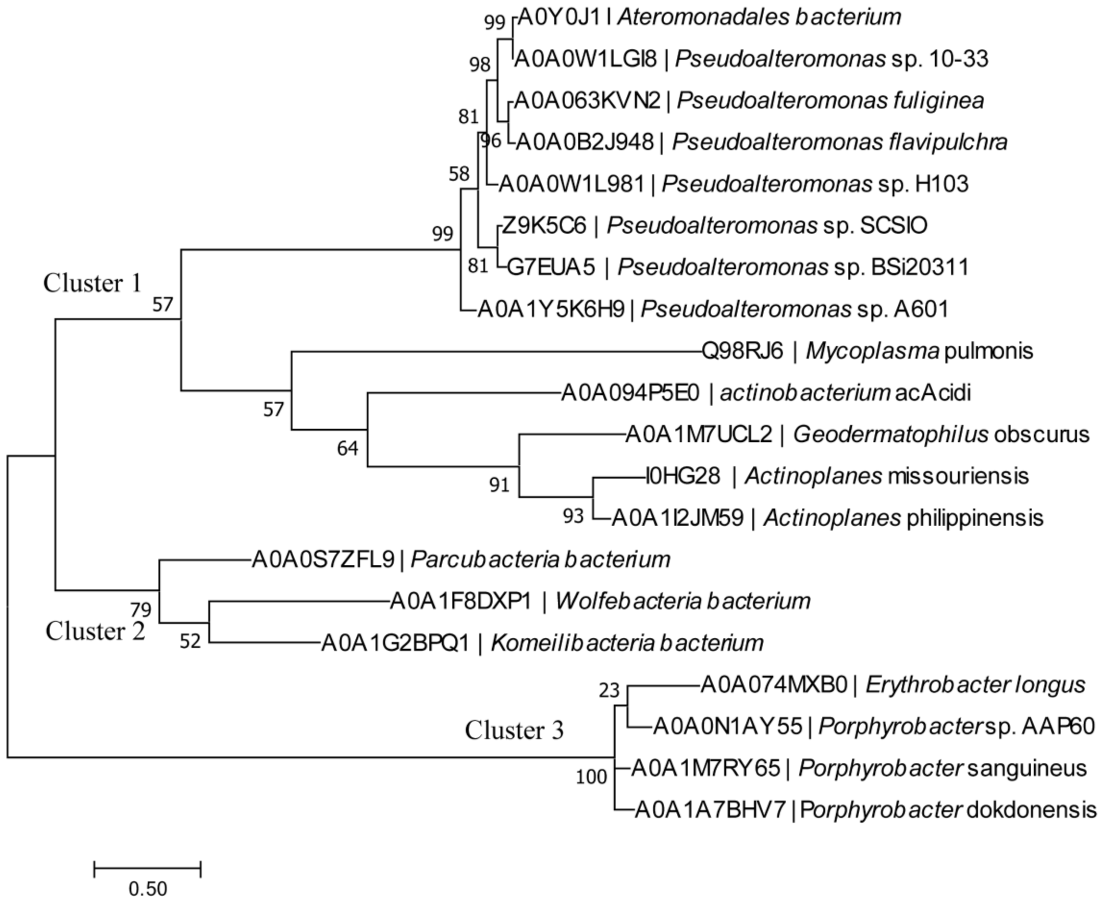

Supplementary Fig. 3. Phylogenetic analysis of transporters with 7 TMHs in bacteria. The Uniprot ID and origin of the proteins are displayed. The phylogenetic tree was constructed using the maximum-likelihood method. The unrooted tree was generated using ClustalW in MEGA 7.0 using SWEET amino acid sequences.

Supplementary Fig. 4.

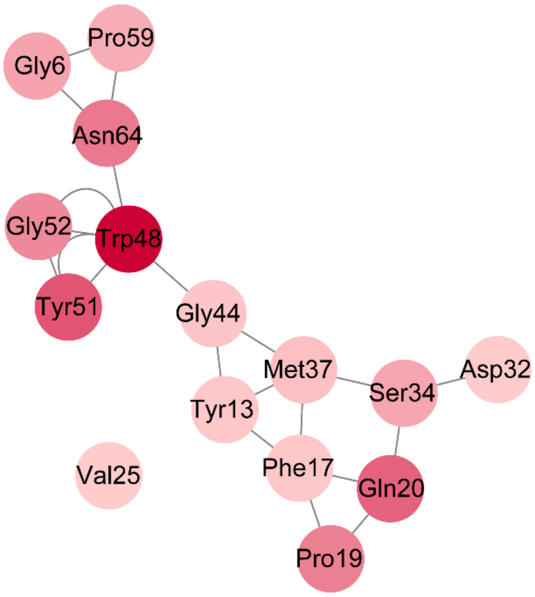

Supplementary Fig. 4. The network cMI with high conservation value. Nodes represent top conserved residues (labeled with position and code) and nodes are colored by conservation from red (higher) to pink (lower). The length of the edges is inversely proportional to MI value (the closest nodes have higher MI).
